# Foresight beats hindsight: The neural correlates underlying motor preparation in the pro‐/anti‐cue paradigm

**DOI:** 10.1002/brb3.663

**Published:** 2017-03-30

**Authors:** Franziska Emmerling, Felix Duecker, Tom A. de Graaf, Teresa Schuhmann, Jos J. Adam, Alexander T. Sack

**Affiliations:** ^1^ Department of Cognitive NeuroscienceFaculty of Psychology and NeuroscienceMaastricht UniversityMaastrichtThe Netherlands; ^2^ Maastricht Brain Imaging CenterMaastrichtThe Netherlands; ^3^ Department of Human Biology and Movement SciencesFaculty of Health, Medicine, and Life SciencesMaastricht UniversityMaastrichtThe Netherlands

**Keywords:** anti‐cue, attention, functional magnetic resonance imaging, priming, proactive, pro‐cue, response inhibition

## Abstract

**Background:**

Human motor behaviors are characterized by both, reactive and proactive mechanisms. Yet, studies investigating the neural correlates of motor behavior almost exclusively focused on reactive motor processes. Here, we employed the *pro‐/anti‐cue* motor preparation paradigm to systematically study proactive motor control in an imaging environment. In this paradigm, either pro‐ or anti‐cues are presented in a blocked design. Four fingers (two from each hand) are mapped onto four visual target locations. Visual targets require a speeded response by one corresponding finger, but, most importantly, they are preceded by visual cues that are congruent (“pro‐cue”), incongruent (“anti‐cue”), or neutral with respect to the responding hand. With short cue‐target intervals, congruence effects are based on automatic motor priming of the correct hand (in case of pro‐cues) or incorrect hand (in case of anti‐cues), generating, respectively, reaction time benefits or reaction time costs relative to the neutral‐cue. With longer cue‐target intervals, slower top‐down processes become effective, transforming early anti‐cue interference into late anti‐cue facilitation.

**Methods:**

We adapted this paradigm to be compatible with neuroimaging, tested and validated it behaviorally—both inside and outside the imaging environment—and implemented it in a whole‐brain functional magnetic resonance imaging study.

**Results and Conclusion:**

Our imaging results indicate that pro‐cues elicited much less neural activation than did anti‐cues, the latter recruiting well‐known cognitive top‐down networks related to attention, response inhibition, and error monitoring/signaling, thereby revealing high‐level influences on proactive motor processes.

## 1 Introduction

Our brains essentially exist for interaction with the environment. As a result, even the simplest tasks relate to many different processes, from low‐level synaptic changes to cognitive, attentional, and context influences. Increasingly, our neuroscientific understanding emphasizes how our brains do not merely respond, but also prepare and anticipate. This enables more efficient processing of, and therefore quicker reactions to, inputs from the environment. Furthermore, preparation enhances decision making in noisy and risky environments by reducing error rates (Schall, 2003). Key to proactive control is a preparatory step before the response is executed. This can be driven by behavioral goals (e.g., favoring speed over accuracy) but also by environmental cues on a trial‐by‐trial base. In the cognitive neuroscience of motor behavior, one such distinction takes the form of *proactive* versus *reactive* mechanisms (Aron, 2011). According to Aron (2011), proactive elements of motor behavior are very prominent in everyday life, even suggesting that the dynamic interaction of proactive and reactive processes underpins almost all real‐life motor actions. Not focusing on the neural correlates of proactive and reactive processes compromises the “ecological validity” of findings (Aron, 2011; Chen, Scangos, & Stuphorn, 2010; Jaffard et al., 2008; Verbruggen, Adams, & Chambers, 2012). Yet, to study such mechanisms in the brain, one requires a robust behavioral paradigm that reliably triggers proactive processes. This is not straightforward given the complexity of influences on motor behavior: For example, proactive inhibitory control varies with context, depending on exogenous and endogenous cues, expectations, and implicit temporal structures in the surroundings (Wardak, Ramanoël, Guipponi, Boulinguez, & Ben Hamed, 2012). While neuroscientific research on proactive control is still in its infancy, a growing number of studies explores the role of various brain regions, such as pre‐supplementary motor area (pre‐SMA), in proactive motor control (Chen et al., 2010; Jaffard et al., 2008; Stuphorn & Emeric, 2012; Wardak, 2011).

**Figure 1:**
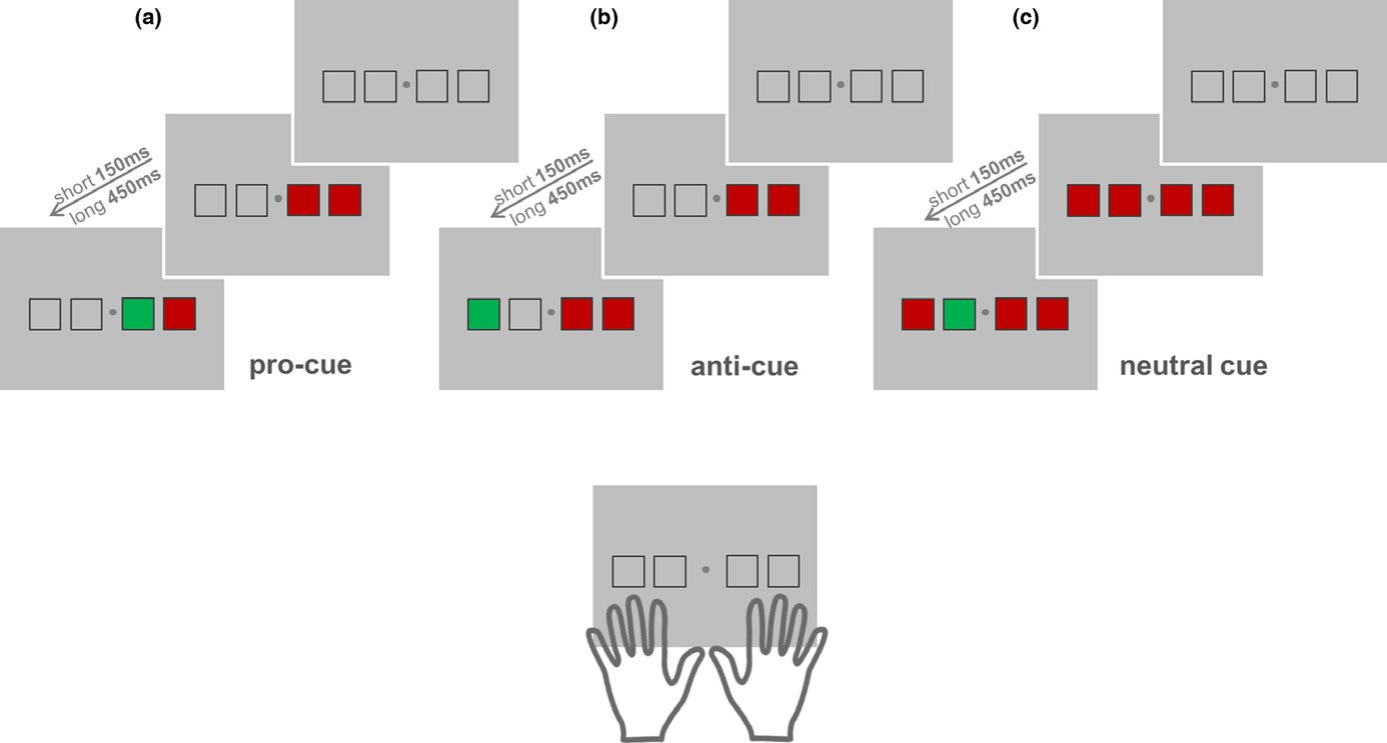
Anti‐cue paradigm. During each trial, two squares were presented on either side of a central fixation dot. The two squares on the right corresponded to the index and middle fingers of the right hand. The two squares on the left corresponded to the index and middle fingers of the left hand. The visual target was a green square. As soon as one of the four squares turned green, participants were asked to press as quickly as possible the corresponding button with the corresponding finger. Cue type. (a) In *pro‐cue trials*, the cue was presented on the same side as the target; thus, the hand on the side of the cue had to be prepared for response. (b) In *anti‐cue trials*, the cue was presented on the side opposite of the target; thus, the hand on the opposite side of the cue had to be prepared for response. (c) In *neutral‐cue trials*, the cue was presented on both sides; thus, there was limited information enabling motor preparation. Cue‐target interval was either *short* (150 ms) or *long* (450 ms). These intervals were selected based on previous research (Adam et al., 2011, 2015) showing reaction time costs induced by anti‐cues (relative to neutral cues) at short delay versus reaction time benefits induced by anti‐cues (relative to neutral cues) at long delay

One paradigm capable of inducing different contexts and temporal structures is the *pro‐/anti‐cue paradigm* (Adam et al., 2011). In this validated choice reaction time task, pro‐ or anti‐cues are presented separately in a blocked design. Four visual target locations are mapped to four motor effectors, namely the index and middle fingers of the right and left hands. Upon onset of a visual target in one of these four locations, participants press a button as quickly as possible with the corresponding finger. Three conditions are differentiated by the preceding appearance of *visual cues* in several of the target locations (see Figure 1). In the *pro‐cue* condition, the responding hand is directly and compatibly primed by visual cues appearing in ipsilateral space (i.e., left‐side cues indicate left hand responses and right‐side cues indicate right hand responses). In contrast, in the *anti‐cue* condition, the responding hand is indirectly primed by visual cues appearing in contralateral space (i.e., left‐side cues indicate right hand responses and right‐side cue indicate left hand responses). In the *neutral‐cue* condition, all four locations are cued, not differentially priming either hand.

Behavioral results show (Adam, Jennings, Bovend'Eerdt, Hurks, & Van Gerven, 2015; Adam et al., 2011) that pro‐cues yield a reaction time benefit compared to neutral cues, while anti‐cues initially result in a reaction time cost caused by the automatic activation of the wrong hand. Importantly, however, this pattern of results only holds for short cue‐target intervals (e.g., 150 ms). Since pro‐cues and anti‐cues are importantly always presented in separate blocks of trials, they are both predictive of the response hand, suggesting that anti‐cues should also lead to performance enhancement given sufficient preparation time. Indeed, with longer cue‐target intervals (e.g., 450 ms), the pro‐cue benefit increases further, but the initial anti‐cue reaction time cost actually reverses into a reaction time benefit that equals that of pro‐cues. So, while anti‐cues initially prime the invalid effectors, given enough time, the motor system can inhibit this wrong, misdirected pre‐activation and correctly prime the contralateral effectors (proactive motor preparation; Adam et al., 2011, 2015).

There are several interesting aspects to this paradigm. Firstly, the onset of a cue triggers a fast, automatic shift of attention to the side of the cue, causing a reflexive activation of the ipsilateral hand due to spatial overlap or congruency between cues and hands (Eimer, Hommel, & Prinz, 1995; Kornblum, Hasbroucq, & Osman, 1990). Secondly, in the anti‐cue condition, this process of automatic response activation by the visual cue primes the *wrong* hand. In other words, the anti‐cue initially induces an error in motor planning, which requires detection, suppression, and correction, for the appropriate button to be pressed in response to the target. Hence, we hypothesized that anti‐cues, relative to pro‐cues, require additional processes related to error signaling and response inhibition. Furthermore, since the target stimulus appears at the side opposite to the cue, an endogenous or top‐down reorientation of attention is needed (Nee, Jonides, & Berman, 2007). While previous work focused on explaining pro‐/anti‐cue behavioral differences in terms of low‐level motor systems (i.e., basal ganglia; Adam et al., 2011), we here hypothesized that high‐level mechanisms of attentional reorientation, error signaling, and response inhibition are also involved. Evidence from related paradigms (e.g., go/nogo, anti‐saccade, finger‐precuing) support this hypothesis (e.g., Adam, Hommel, & Umiltà, 2003, 2005; Chambers, Garavan, & Bellgrove, 2009; Munoz & Everling, 2004; Swick, Ashley, & Turken, 2011).

In the current experiment, we explored for the first time the high‐level whole‐brain neural correlates underlying motor preparation in pro‐ and anti‐cue conditions using functional magnetic resonance imaging (fMRI). In particular, we tested the hypothesis that a facilitation in mental processing might lead to the recruitment of less brain activation in terms of spatial distribution. In other words, we hypothesized, that pro‐cues show much less neural activation than anti‐cues, due to the congruency (spatial overlap) between cues and response hand in pro‐cue but not anti‐cue conditions, creating direct and automatic facilitation of the responding hand. This conceptualization is in line with dual‐route models of response selection (e.g., Kornblum et al., 1990), which posit two response selection routes: (1) a bottom‐up, automatic route, which enables stimulus‐driven, fast, and direct response selection and (2) a top‐down, effortful route, which enables indirect and deliberate, task‐dependent response selection. The latter route allows for flexibility in choosing actions by endogenous or top‐down control of stimulus‐driven activation. In this study, we aimed to characterize the neural areas associated with reflexive, bottom‐up proactive control and those associated with intentional, top‐down proactive control, driven, respectively, by pro‐cues and anti‐cues.

We first adapted the pro‐cue/anti‐cue paradigm to be compatible with fMRI. As it was shown previously that the validity of cognitive paradigms might suffer from being introduced to an imaging environment (van Maanen, Forstmann, Keuken, Wagenmakers, & Heathcote, 2016), we validated the paradigm on the behavioral level both outside and inside the imaging environment. Finally, we explored and contrasted the involvement of cognitive brain networks in pro‐cue and anti‐cue conditions. Looking ahead, we could indeed reveal a much stronger and more extensive neural activation pattern in terms of spatial distribution during anti‐cue compared to pro‐cue performance, including neural areas known to be involved in attention (shifting), error signaling, and response inhibition. Our results (1) emphasize the plethora of neural influences on even the simplest motor response task; (2) delineate neural correlates underlying bottom‐up and top‐down modes of proactive motor behavior; and (3) can guide further investigation of this particularly valuable proactive action paradigm.

## 2 Materials and Methods

### 2.1 Participants

Twenty‐two right‐handed healthy volunteers (mean age = 29, *SD* = 3.04) with normal or corrected‐to‐normal vision participated in the *behavioral experiment 1*. They gave their written informed consent prior to participating, and were paid for taking part. The study was approved by the local Ethical Committee of the Faculty of Psychology and Neuroscience, Maastricht University. A subset of participants from experiment 1, who showed the basic cross‐over reaction time effect, were invited for experiment 2. Eighteen participants (mean age = 28, *SD* = 3.45) took part in the *fMRI experiment 2*.

### 2.2 Stimuli, task, and design

We used an adapted version of the pro‐/anti‐cue paradigm (Adam et al., 2011; see Figure 1). Cue‐type as well as cue‐target intervals were varied (see Figure 1 for details). As a first research question, we assessed whether previous behavioral findings are replicable in our adapted behavioral task (experiment 1) and inside the MR environment (experiment 2). Indeed, we confirmed this behavioral pattern both prior to scanning and during the fMRI measurement (see Section 3).

In both experiments, there were six conditions in total, in a cue type (pro‐cue, anti‐cue, neutral‐cue) by cue‐target interval (short, long) within‐subject design. Cues preceded the target by either 150 ms or 450 ms, depending on the cue‐target interval, and remained visible until the target disappeared. Targets were presented for 500 ms. The inter ‐trial interval varied between 1,000 and 3,000 ms (locked to the repetition time/TR). Targets were green and cues were red, all presented on a gray background. No error feedback was provided for participants.

Stimulus presentation was organized in a block design. Each experimental session consisted of four runs. Two runs included exclusively pro‐cue blocks; two runs included exclusively anti‐cue blocks. So, pro‐ versus anti‐cue blocks were strictly separated between fMRI runs. Other conditions (short vs. long cue‐target intervals and neutral‐cue trials) were mixed within each run, but still separated in blocks of six trials each. Each run contained 16 such blocks. These blocks each contained trials with only short or only long cue‐target intervals, and with exclusively informative (pro or anti) or neutral cues. In summary, pro‐/anti‐cue trials were separated into different runs, and trials with short versus long cue‐target intervals were separated into different blocks within runs. The four runs were presented in fully randomized order.

There were, therefore, two adaptations to the original pro‐/anti‐cue paradigm: (1) inclusion of only two cue‐target intervals instead of a full range (100, 150, 250, 450, and 850 ms) and (2) grouping trials in blocks of all experimental conditions instead of interleaving trials. It was an empirical question as to whether the selected intervals would fully capture the “cross‐over” effect from reaction time costs to benefits in the anti‐cue condition, or whether, for instance, temporal idiosyncrasies, that is, inter‐individual variability, in attention/motor systems would necessitate a more extensive individual calibration of behavioral response curves. All of this predicated on the untested assumption that the required reaction time costs and benefits would arise from this adapted paradigm at all, given the new block design. These questions were addressed prior to fMRI in behavioral experiment 1 and again inside the MR environment in experiment 2. In each experimental session, 384 trials were recorded in total (96 pro‐cue trials, 96 anti‐cue trials, 192 neutral‐cue trials split between pro‐cue runs and anti‐cue runs). It took approximately 40 min to complete the task. Stimuli were presented using Presentation software (Neurobehavioral Systems, Inc., Albany, USA).

### 2.3 Technical details and fMRI acquisition

In experiment 1, responses were collected using a generic keyboard; in the MRI scanner, responses were collected with a standard MR‐compatible button box (Current Designs, 8‐button response device, HHSC‐2x4‐C, Philadelphia, USA). With a 3 Tesla Siemens Prisma MR scanner, structural (high resolution T1‐weighted MPRAGE; isotropic voxel resolution 1 × 1 × 1 mm^3^; 192 sagittal slices) and functional whole‐brain (Gradient‐Echo‐EPI‐sequence; multiband acceleration factor of 2; TR = 1,000 ms; TE = 29 ms; FOV = 216 mm; flip angle = 62°; distance factor = 15%; 603 volumes per run) images were acquired. Thirty‐two oblique transversal slices of 3.0 × 3.0 × 3.0 mm voxels, tilted 30° relative to the anterior‐posterior commissural plane, were obtained to avoid signal dropout in frontal areas (Deichmann, Gottfried, Hutton, & Turner, 2003).

### 2.4 Behavioral data analysis of experiment 1 and 2

The statistical analysis of the behavioral data was restricted to reaction times, yet, it is important to note that participants performed the task with high levels of accuracy in both experiments (experiment 1: 95%; experiment 2: 97%; equal reaction time pattern independent of errors). One participant was removed from the behavioral data analysis of the fMRI session (experiment 2) due to failure to use the correct response buttons.^1^ For the analysis of reaction time data, we first excluded all incorrect trials and removed outliers according to the 1.5 inter‐quartile range criterion for each condition in each participant. Then, mean reaction time data were submitted to repeated‐measures analyses of variances (ANOVAs). For all repeated‐measures ANOVAs, we report the multivariate test statistics (Pillai's trace) and we used the customary significance level of *p *<* *.05. Post hoc paired *t* tests were used to explore simple effects when appropriate, as indicated by significant interactions, and *p*‐values are reported after Bonferroni correction, performed for both experiments separately as they were considered to be independent. All analyses were performed using IBM SPSS Statistics version 21 (IBM, Armonk, NY, USA).

### 2.5 FMRI analysis of experiment 2

Data analyses were performed using Brain Voyager QX 2.8.2 (Brain Innovation BV, Maastricht, the Netherlands). Preprocessing included three‐dimensional motion correction (as implemented in Brain Voyager QX with trilinear/sinc interpolation and intrasession alignment to the first functional volume recorded after the individual anatomical scan), cubic spline slice scan time correction, and the application of a temporal high‐pass filter (general linear model [GLM] with Fourier basis set of three cycles sine/cosine per run including linear trend removal). Images were coregistered to the individual anatomical scans and normalized to Talairach stereotaxic space (Talairach & Tournoux, 1988). Volume time courses were spatially smoothed using a 6 mm full width half maximum Gaussian kernel.

Random effects group analyses were performed. A GLM was defined in order to analyze specific task‐related activation patterns for the different conditions. The GLM included eight predictors (pro‐cue and neutral‐cue short, pro‐cue and neutral‐cue long, anti‐cue and neutral‐cue short, anti‐cue and neutral‐cue long). Note that neutral‐cue predictors were thus differentiated based on whether they occurred in pro‐cue runs or in anti‐cue runs, to serve as dedicated contrasts for a first‐level analysis (pro‐ vs. neutral‐cue and anti‐ vs. neutral‐cue, see below). Motion parameters were included as confound predictors in the regression analysis. Statistical maps were created using a threshold of *p* < .001 corrected for multiple comparisons by means of cluster threshold level estimation analysis (1,000 Monte Carlo simulation iterations; Forman et al., 1995).

In a first‐level analysis, all experimental pro‐ and anti‐cue conditions were contrasted with their corresponding neutral‐cue condition, resulting in four baseline‐corrected conditions for second‐level analysis: namely *pro‐cue short*,* pro‐cue long*,* anti‐cue short*,* anti‐cue long*. This first‐level analysis was validated by direct contrasts between different implementations of neutral‐cue trials, which did not reveal any significant results. We performed three main second‐level analyses on the resulting data. First, collapsing over cue‐target intervals, we contrasted pro‐cue versus anti‐cue trials. Then, we contrasted pro‐cue versus anti‐cue trials for each cue‐target interval. And finally, within pro‐ and anti‐cue runs, we contrasted the short versus long cue‐target interval.

## 3 Results

### 3.1 Experiment 1

**Figure 2:**
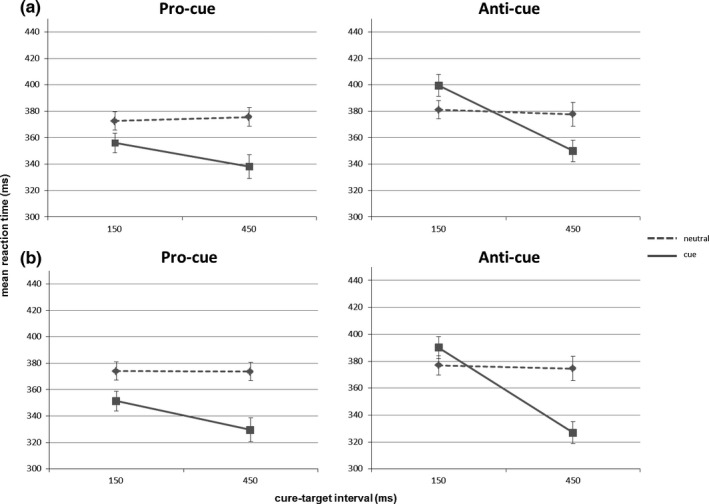
Behavioral effect of cuing. (a) Behavioral effect of cuing in the behavioral experiment 1. (b) Behavioral effect of cuing in the imaging experiment 2. *x*‐axis: cue‐target interval in ms; *y*‐axis: mean reaction time in ms

**Figure 3:**
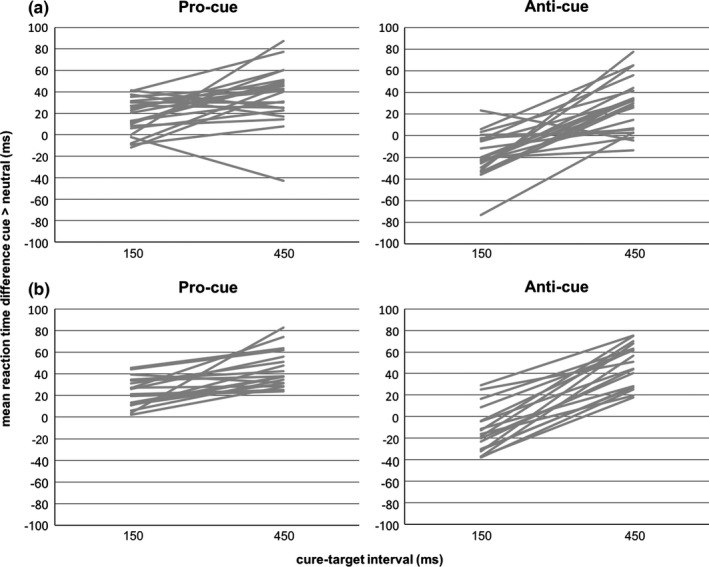
Behavioral effect of cuing on single subject level. (a) Behavioral effect of cuing in the behavioral experiment 1. (b) Behavioral effect of cuing in the imaging experiment 2. Differential reaction time: mean reaction time after neutral‐cues—mean reaction time after pro‐/anti‐cues; positive values indicate reaction time benefits, negative values indicate reaction time costs

We adapted the established pro‐/anti‐cue paradigm to make it compatible with block‐design neuroimaging. Not only for the purposes of the current experiment, but also for future work, it was relevant to know: (1) do the relevant behavioral results (indicative of condition‐specific proactive preparation) accrue even with all condition cells blocked and (2) do the relevant behavioral results reliably accrue with only two (noncalibrated) cue‐target intervals? In both cases, the relevant behavioral results would consist of (1) reaction time benefits from pro‐cues with short cue‐target interval and reaction time costs from anti‐cues with short cue‐target interval and (2) reaction time benefits from both pro‐ and anti‐cues with long delays. In Figure 3, we present the results not only for the group mean (also shown in Figure 2) but also for individual participants, allowing visual inspection of the consistency of behavioral effects across the sample.

A repeated‐measures ANOVA on mean reaction times (Table 1) with condition (pro, anti), cue type (informative, neutral), and cue‐target interval (150, 450 ms) as within‐subject factors revealed that all main effects and interactions were significant (all *p*‐values < .01). The important significant three‐way interaction, *F*(1, 21) = 8.918, *p* < .01, $\eta_{p}^{2}$ = .298, was further explored with follow‐up ANOVAs for the pro‐cue and anti‐cue condition separately. Because of this higher order interaction, we did not further consider the remaining interactions and main effects.

**Table 1:** Mean reaction times (in ms) and standard error of the mean for each experimental condition in both experiments

Cue‐target interval	Experiment 1		Experiment 2	
	150	450	150	450
Pro‐cue	356 ± 7.5	338 ± 9.0	348 ± 5.6	327 ± 5.1
Neutral‐cue within pro‐cue condition	373 ± 6.9	376 ± 7.0	370 ± 5.8	370 ± 7.3
Anti‐cue	400 ± 8.3	350 ± 8.2	388 ± 8.7	325 ± 6.1
Neutral cue: within anti‐cue condition	381 ± 7.0	378 ± 9.0	373 ± 7.5	371 ± 7.1

A repeated‐measures ANOVA on mean reaction times in the pro‐cue condition with cue type (pro‐cue, neutral‐cue) and cue‐target interval (150, 450 ms) as within‐subject factors revealed significant main effects of cue type, *F*(1, 21) = 55.673, *p* < .001, $\eta_{p}^{2}$ = .726, and cue‐target interval, *F*(1, 21) = 5.620, *p* < .05, $\eta_{p}^{2}$ = .211, and a significant interaction between these factors, *F*(1, 21) = 11.805, *p* < .005, $\eta_{p}^{2}$ = .360. Post hoc paired *t* tests showed that this result was driven by two observations. To begin with, pro‐cues led to decreased reaction times at short, *t*(21) = 4.666, *p* < .001, *d*_*z*_ = 0.995, but also at long cue‐target intervals, *t*(21) = 6.640, *p* < .001, *d*_*z*_ = 1.416. Note that—as indicated by the significant interaction reported above—this benefit was significantly more pronounced at long compared to short cue‐target intervals. These results clearly indicate that pro‐cues effectively boosted performance at both cue‐target intervals, showing a fast, automatic activation of the correct response hand at the short cue‐target interval and an additional benefit of endogenous cueing processes at the long cue‐target interval.

A repeated‐measures ANOVA on mean reaction times in the anti‐cue condition with cue type (anti‐cue, neutral‐cue) and cue‐target interval (150, 450 ms) as within‐subject factors revealed a significant main effect of cue‐target interval, *F*(1, 21) = 64.935, *p* < .001, $\eta_{p}^{2}$ = .756, and no significant main effect of cue type, *F*(1, 21) = 2.011, *p* > .10, $\eta_{p}^{2}$ = .087, due to the significant interaction between cue type and cue‐target interval, *F*(1, 21) = 45.330, *p* < .001, $\eta_{p}^{2}$ = .683. Indeed, post hoc paired *t* tests revealed the predicted pattern of reaction time differences for both cue‐target intervals. For the short cue‐target interval, anti‐cues were associated with significantly increased reaction times compared to neutral cues, *t*(21) = 4.353, *p* < .005, *d*_*z*_ = 0.928, thus a reaction time cost, consistent with the idea that anti‐cues lead to automatic activation of the wrong response hand. In contrast, anti‐cues led to decreased reaction times compared to neutral‐cues at long cue‐target intervals, *t*(21) = 5.311, *p* < .001, *d*_*z*_ = 1.132; thus, a reaction time benefit demonstrating that this automatic process can be overruled to enhance performance, given sufficient time. Lastly, the reaction time benefit observed for the long cue‐target interval in the anti‐cue condition did not significantly differ in magnitude from the reaction time benefit in the long cue‐target interval in the pro‐cue condition (no significant difference: *t*(21) = 1.536, *p* > .80, *d*_*z*_ = 0.327).

### 3.2 Experiment 2

#### 3.2.1 Behavioral results

The behavioral data from the fMRI experiment were analyzed in the same way as the data from experiment 1. We replicated all our behavioral findings in the scanner environment, showing that the paradigm is robust to this context change and can result in a distinct pattern of benefits and costs in the pro‐ and anti‐cue conditions as hypothesized. In Figure 3, we present the results not only for the group mean (also shown in Figure 2) but also for individual participants, allowing visual inspection of the consistency of behavioral effects across the sample.

A repeated‐measures ANOVA on mean reaction times with condition (pro, anti), cue type (informative, neutral), and cue‐target interval (150, 450 ms) as within‐subject factors revealed that all main effects and interactions were significant (all *p*‐values < .005). Again, the significant three‐way interaction, *F*(1, 16) = 40.098, *p* < .001, $\eta_{p}^{2}$ = .715, was further explored with follow‐up ANOVAs for the pro‐cue and anti‐cue conditions, separately.

A repeated‐measures ANOVA on mean reaction times in the pro‐cue condition with cue type (pro‐cue, neutral‐cue) and cue‐target interval (150, 450 ms) as within‐subject factors revealed significant main effects of cue type, *F*(1, 16) = 141.689, *p* < .001, $\eta_{p}^{2}$ = .899, and cue‐target interval, *F*(1, 16) = 13.955, *p* < .005, $\eta_{p}^{2}$ = .466, and a significant interaction between these factors, *F*(1, 16) = 18.759, *p* < .005, $\eta_{p}^{2}$ = .540. Post hoc paired *t* tests showed again that this result was due to decreased reaction times for pro‐cues compared to neutral‐cues at short, *t*(16) = 6.784, *p* < .001, *d*_*z*_ = 1.645, and long, *t*(16) = 10.340, *p* < .005, *d*_*z*_ = 2.509, cue‐target intervals and, leading to the significant interaction, this benefit was significantly more pronounced at long compared to short cue‐target intervals.

A repeated‐measures ANOVA on mean reaction times in the anti‐cue condition with cue type (anti‐cue, neutral‐cue) and cue‐target interval (150, 450 ms) as within‐subject factors revealed significant main effects of cue type, *F*(1, 16) = 15.292, *p* < .005, $\eta_{p}^{2}$ = .489, and cue‐target interval, *F*(1, 16) = 66.652, *p* < .001, $\eta_{p}^{2}$ = .806, and a significant interaction between these factors, *F*(1, 16) = 150.432, *p* < .001, $\eta_{p}^{2}$ = .904. Post hoc paired *t* tests revealed again the predicted pattern of reaction time differences for both cue‐target intervals. Anti‐cues were associated with significantly increased reaction times compared to neutral‐cues for short cue‐target intervals, *t*(16) = 3.376, *p* < .05, *d*_*z*_ = 0.819, and decreased reaction times compared to neutral‐cues at long cue‐target intervals, *t*(16) = 9.955, *p* < .005, *d*_*z*_ = 2.414. Lastly, the reaction time benefit observed for the long cue‐target interval in the anti‐cue condition did not significantly differ in magnitude from the reaction time benefit in the long cue‐target interval in the pro‐cue condition (no significant difference: *t*(16) = 0.830, *p* = 1.00, *d*_*z*_ = 0.201).

In a final analysis, we took advantage of the fact that all participants in the fMRI experiment also took part in the preceding behavioral experiment. We could thus directly compare the magnitude of benefits and costs from both experiments by submitting reaction time differences between informative and neutral‐cues to a repeated‐measures ANOVA with experiment (behavioral, fMRI), task (pro, anti), and cue‐target interval (150, 450 ms) as within‐subject factors. This analysis confirmed the findings reported above as indicated by a significant interaction between task and cue‐target interval, *F*(1, 16) = 46.972, *p* < .001, $\eta_{p}^{2}$ = .746, and, more importantly, provided no evidence that benefits and costs were statistically distinguishable across sessions, that is, the three‐way interaction between experiment, task, and cue‐target interval was not significant, *F*(1, 16) = 1.012, *p* > .30, $\eta_{p}^{2}$ = .060, just as the main effect of experiment and the remaining interactions involving this factor (all *p*‐values > .10). This is relevant, since it suggests that the paradigm in current form is not only replicable, but also robust with respect to order effects or learning. This may open up opportunities for neuroimaging studies with multiple sessions and additional manipulations.

#### 3.2.2 FMRI results

While previous work on the pro‐/anti‐cue paradigm demonstrated low‐level motor system involvement, we here asked whether high‐level cognitive processes are also at play. As outlined in the Method section, we first contrasted pro‐/anti‐cue conditions with their neutral‐cue counterparts, as a form of baseline correction in a first‐level analysis. On the resulting data, we performed three main second‐level analyses; overall pro‐cue versus anti‐cue activations, pro‐cue versus anti‐cue separately for both cue‐target intervals, and short versus long cue‐interval separately for both cue types. We here list the resulting activations (reported numerically in Table 2).

**Table 2:** FMRI statistics

Region		Talairach coordinates			SizeVoxel	*t*
		*x*	*y*	*z*		
Pro‐cue short + long > neutral‐cues						
Middle frontal gyrus BA6	R	38	0	39	1,887	6.72
Inferior parietal lobe BA40	L	−55	−49	40	2,023	6.07
Inferior parietal lobe BA40	L	−36	−47	37	430	4.84
Occipital Lobe BA18	R	25	−70	−8	830	−5.79
Occipital Lobe BA18	R	15	−87	19	453	−5.34
Anti‐cue short + long > neutral‐cues						
Insular cortex BA13	R	34	14	8	2,421	6.87
Insular cortex BA13	L	−39	12	6	1,350	6.46
Middle frontal gyrus BA9	R	38	44	28	867	6.95
Middle frontal gyrus BA6	R	29	−5	49	6,805	8.15
Middle frontal gyrus BA9	L	−40	27	33	468	5.82
Middle frontal gyrus BA6	L	−27	−7	53	4,035	6.23
Medial frontal gyrus BA23	R	4	10	45	540	5.19
Cingulate gyrus BA23		0	−23	26	1,255	5.90
Precuneus BA7	R	14	−62	45	17,089	6.58
Superior Parietal Lobe BA7	L	−23	−61	44	25,014	8.21
Pro‐cue short > pro‐cue long^a^						
Superior frontal gyrus BA9	L	−13	62	30	576	6.23
Precentral gyrus BA44	R	59	7	8	465	−5.39
Inferior frontal gyrus BA44	L	−50	3	20	636	−5.98
Paracentral lobe BA6	R	11	−30	59	1,132	−5.07
Precentral lobe BA4	R	15	−31	59	1,409	−5.97
Inferior parietal lobe BA40	L	−47	−38	30	1,229	−5.07
Precuneus BA7	R	24	−63	39	316	−4.88
Precuneus BA7	L	−18	−59	47	1,046	−5.07
Precuneus BA7	R	8	−51	52	803	−4.78
Anti‐cue short > anti‐cue long^a^						
Lentiform nucleus/putamen	R	28	−15	8	619	5.41
Superior frontal gyrus BA10	R	17	54	16	651	5.13
Postcentral gyrus BA3	R	28	−26	43	465	5.74
Cingulate gyrus BA24	R	7	−15	42	746	5.63
Pro‐cue short > anti‐cue short^a^						
Cerebellum	R	31	−53	−11	306	−4.74
Insular cortex BA13	R	39	5	11	750	−5.03
Inferior parietal lobe BA40	R	43	−34	40	561	−4.76
Precuneus BA7	L	−13	−69	52	347	−5.04
Pro‐cue long > anti‐cue long^a^						
Superior temporal gyrus BA38	R	32	8	−22	956	5.37
Middle frontal gyrus BA11	R	38	42	−12	728	5.43
Lentiform nucleus/putamen	R	29	−20	13	1,989	6.42
Paracentral lobe BA6	R	7	−24	50	3,952	7.25
Postcentral gyrus BA3	R	24	−28	48	417	5.65
Cerebellum	R	20	−69	−10	1,182	−6.12
Precuneus BA7	L	−4	−68	48	517	−5.84

Talairach coordinates, size and *t*‐value for all identified clusters. Cluster sizes are reported in voxel.

a Corrected for neutral‐cues.

##### Overall pro‐cue and anti‐cue versus neutral‐cue

**Figure 4:**
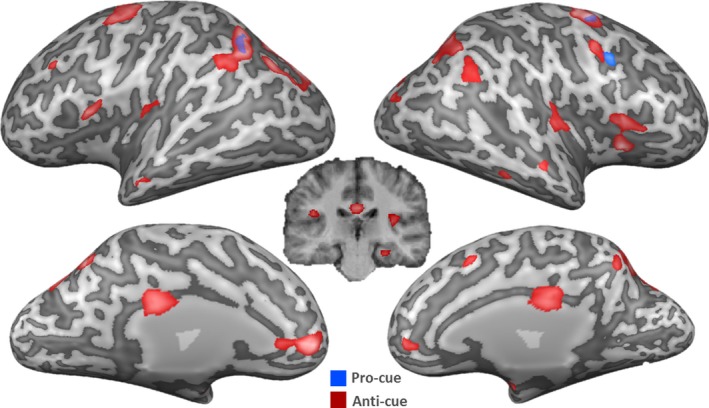
Activation of brain networks differentially involved in the anti‐ and pro‐cue conditions. All pro‐cue trials depicted in blue; all anti‐cue trials depicted in red. All conditions compared to their respective neutral condition. RFX GLM *N* = 18 *p* < .001 cluster level threshold corrected. Right hemisphere depicted on the right side. Significant activation projected onto an inflated surface of a single subject's brain

The contrast map for overall pro‐cue and anti‐cue blocks versus their respective neutral‐cue conditions is presented in Figure 4. Collapsed over cue‐target intervals, pro‐cue related activity was restricted to two key areas, namely the right middle frontal and bilateral inferior parietal regions. Anti‐cues, on the other hand led to substantially more activation, that is, higher bilateral activation in insular and middle frontal cortex, as well as the right medial frontal gyrus, the cingulate gyrus, the right precuneus, and the left superior parietal lobe.

##### Pro‐cue versus anti‐cue as a function of cue‐target interval

We performed this same contrast separately per cue‐target interval condition, but nevertheless included the overall contrast since it included twice the data and thereby increased statistical power.

**Figure 5:**
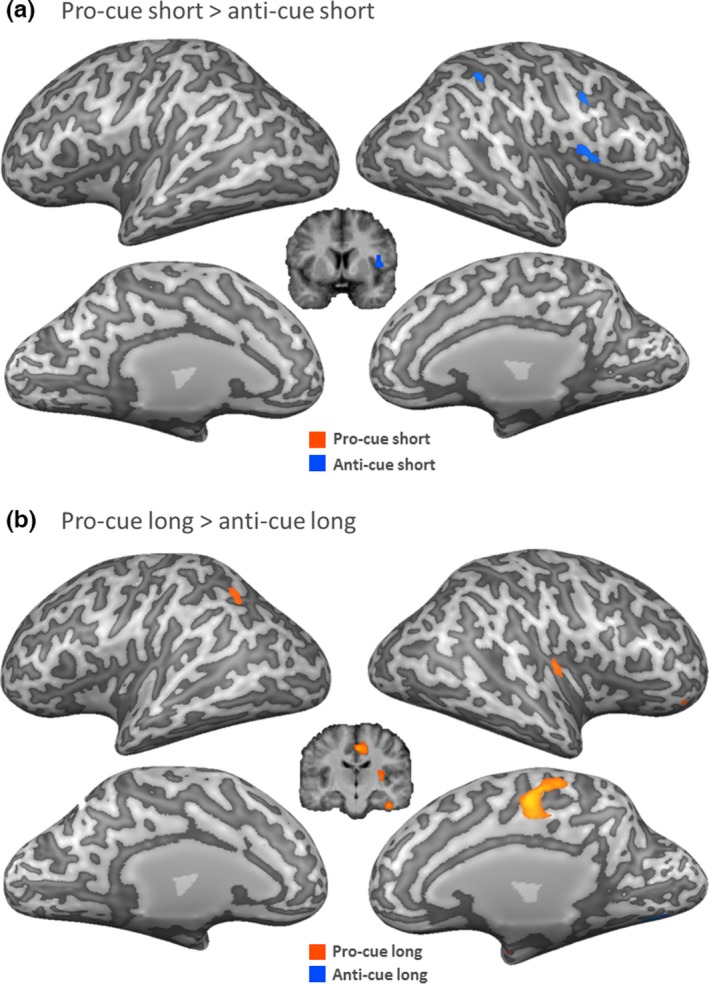
Group level activation within time‐conditions. (a) Direct contrast between conditions, pro‐cue short and anti‐cue short. (b) Direct contrast between conditions, pro‐cue long and anti‐cue long. All conditions compared to their respective neutral condition. RFX GLM *N* = 18 *p* < .001 cluster level threshold corrected. Right hemisphere depicted on the right side. Significant activation projected onto an inflated surface of a single subject's brain. Contrasts corrected for neutral‐cues

The results of the pro‐cue > anti‐cue contrasts separately per cue‐target interval are shown in Figure 5. For short intervals (pro‐cue short > anti‐cue short, Figure 5a), anti‐cues were associated with increased activity in the right cerebellum, the right insular cortex, the right inferior parietal lobe, and the left precuneus. No clusters more active with pro‐cues survived thresholding. For long intervals (pro‐cue long > anti‐cue long, Figure 5b), pro‐cues induced stronger activations in the right superior and middle frontal gyri, the right putamen, the right paracentral lobe, and the right postcentral gyrus. Anti‐cues were associated with higher activity in the right cerebellum and the left precuneus.

##### Short versus long cue‐target interval for pro‐cues and anti‐cues

**Figure 6:**
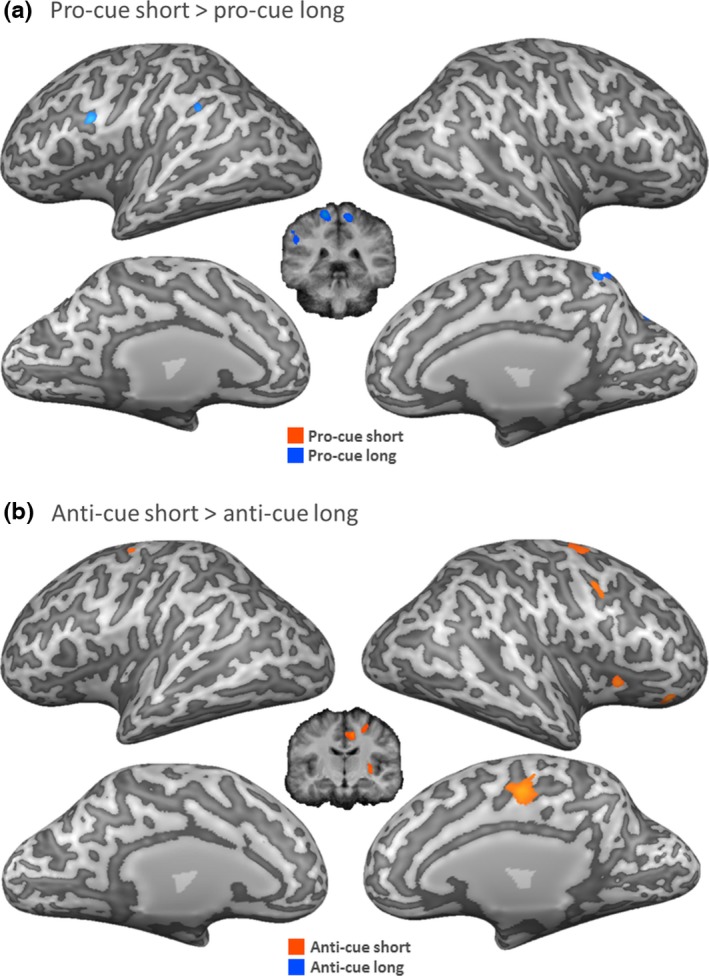
Group level activation within cue conditions. (a) Direct contrast between conditions, pro‐cue short and pro‐cue long. (b) Direct contrast between conditions, anti‐cue short and anti‐cue long. All conditions compared to their respective neutral condition. RFX GLM *N* = 18 *p* < .001 cluster level threshold corrected. Right hemisphere depicted on the right side. Significant activation projected onto an inflated surface of a single subject's brain. Contrasts corrected for neutral‐cues

Results of the final analysis are displayed in Figure 6, contrasting cue‐target intervals with pro‐cues (pro‐cue short > pro‐cue long, Figure 6a) or anti‐cues (anti‐cue short > anti‐cue long, Figure 6b). With pro‐cues, the short cue‐target interval resulted in increased activation in the left superior frontal gyrus, while the long cue‐target interval showed increased activity in the right precentral gyrus, left inferior frontal gyrus, right paracentral lobe, left inferior parietal lobe, and the bilateral precuneus. Significant activation for the anti‐cue trials with a short cue‐target interval was observed in exclusively right‐lateralized regions, that is, in the putamen, the superior frontal gyrus, the postcentral gyrus, and the cingulate gyrus.

No clusters more active in the long‐interval anti‐cue condition as opposed to the short‐interval condition survived thresholding. The behavioral measures nicely capture a sequence of cognitive events through the long‐ versus short‐interval RT dissociation. FMRI, however, captures all those processes and more, and the only way to extract meaningful information is to directly compare different conditions. The lack of significant clusters in the contrast (anti‐cue long > anti‐cue short), should most definitely not be taken to indicate that the long‐interval anti‐cue condition did not correlate to an extensive and strong activation response throughout many regions in the brain. To see that response, one should visualize the contrast with the long‐interval anti‐cue condition and the brain at rest, but the result would be so broad that it will not be meaningful to interpret.

In sum, across the analyses, several main results converge. Firstly, a collective of well‐known cognitive network nodes are more active in the pro‐/anti‐cue conditions as compared to neutral blocks. We discuss these clusters in more detail below. Secondly, confirming our hypothesis, overall there is substantially more activation in anti‐cue blocks than in pro‐cue blocks. Thirdly, activations resulting from the various contrasts mostly converge.

## 4 Discussion

In this study, we adapted the pro‐/anti‐cue task to be compatible with neuroimaging and validated it behaviorally prior to, and again during, the fMRI measurements. In both behavioral data sets, we robustly replicated the key behavioral characteristics of this paradigm. We then explored the neural correlates of motor preparation by means of fMRI. The neural manifestation of motor preparation was examined with respect to different contexts (pro‐cues versus anti‐cues) and different temporal structures (short vs. long cue‐target intervals). Results demonstrated widespread involvement of well‐known cognitive networks. Below, we relate our results to previous research to begin disentangling the various higher order processes involved in proactive motor preparation based on pro‐ and anti‐cues.

### 4.1 Attention mechanisms

The present paradigm has some aspects that are comparable to the classical Posner cueing paradigm (Posner, 1980; Posner, Snyder, & Davidson, 1980). Unsurprisingly, both anti‐ and pro‐cue trials led to activation of several brain areas commonly associated with attentional control (Corbetta & Shulman, 2002). These clusters were found in parietal and frontal cortex, overlapping with the typical core nodes of the dorsal and ventral attention network. Matching the characteristics of the behavioral paradigm, dorsal attention network nodes in the posterior parietal cortex and the precentral sulcus (presumably the frontal eye field) were activated, most likely reflecting the orientation of attention toward the location of the upcoming target stimulus, which was shown in many previous neuroimaging studies using traditional spatial orienting paradigms (Doricchi, Macci, Silvetti, & Macaluso, 2010; Hopfinger, Buonocore, & Mangun, 2000; Kincade, Abrams, Astafiev, Shulman, & Corbetta, 2005). Similarly, ventral attention network nodes in inferior parietal and frontal cortex were activated, in particular, during anti‐cue trials, most likely reflecting the control over “distracting” anti‐cues and the reorientation toward the target stimulus. Previous work has generally attributed a circuit‐breaker function to the ventral attention network, that is recruited when unexpected or behaviorally relevant stimuli are detected, so that attentional reorienting can be initiated (Corbetta, Kincade, Ollinger, McAvoy, & Shulman, 2000; Kincade et al., 2005). Moreover, the temporo‐parietal junction, a core node of this network, has recently been implicated in contextual updating (Geng & Vossel, 2013); thus, fitting the task demands of the anti‐cue condition with its incongruent mapping of cue and target location. Taken together, the observed activation pattern is consistent with the attentional demands of the pro‐/anti‐cue task, which not only taps into motor control processes but also requires attentional processes associated with anticipation and detection of visual stimuli at cued (congruent) and non‐cued (incongruent) locations.

At this point, it is useful to consider the possible role of eye movements in our paradigm. Huestegge and Adam (2011) showed that the execution of saccades during the cue‐target interval can interfere with manual response preparation. That is, they reported evidence for a cross‐modal response interference effect when the cue triggered a saccade in the direction opposite to the to‐be‐prepared response (hand). Similarly, anti‐cues in this study, but not pro‐cues, exhibit spatial incongruency between cues (which may trigger saccades) and response hand, and thus might be sensitive to cross‐modal interference. Consequently, anti‐cue costs observed with short cue‐target intervals might be due not only to covert attention shifts but also to overt eye movements, which have been shown to recruit largely overlapping fronto‐parietal circuits, including the frontal eye fields and intraparietal areas (Corbetta et al., 1998; De Haan, Morgan, & Rorden, 2008). Interestingly, Huestegge and Adam (2011) also reported that the actual execution of eye movements strongly depended on the length of the cue‐target interval. Although the longer cue‐target interval of 500 ms triggered saccades on a substantial portion of the trials (44.2%), the shorter cue‐target interval of 100 ms triggered saccades only on a minority of the trials (6.4%). This finding seems to suggest a limited contribution of eye movements to the observed anti‐cue cost with the short cue‐target interval.

### 4.2 Response inhibition

Anti‐cue trials involved substantial activation in the anterior insular cortex (BA13). This activation was apparent in the overall contrast anti‐cues versus pro‐cues (Figure 4), and mirrored in the contrast map of anti‐cue trials with short cue‐target interval compared to pro‐cue trials with short cue‐target interval (Figure 6). Anterior insular activation has repeatedly been associated with response restraint and response cancellation (Dambacher et al., 2014a, 2014b, 2014c; Swick et al., 2011). It should be emphasized that the reported insular activation extended very prominently into the inferior frontal cortex, which is classically associated with motor response inhibition (Aron, Robbins, & Poldrack, 2004; Chambers et al., 2006, 2007).

In addition, the activity in the cerebellum was elevated in anti‐cue trials compared to pro‐cue trials (both in the short and long cue‐target intervals), which accords with recent evidence pointing to a critical role of the cerebellum in response inhibition (Picazio & Koch, 2015).

Anti‐cue blocks require inhibitory resources to suppress the effector ipsilateral to the cue before initiating the response contralateral to the cue. This process might be especially demanding when time between the anti‐cue and the imperative target is very short. Due to the block design, participants were aware of the short interval on these trials, possibly resulting in enhanced task engagement, causing higher activation in insular regions for short versus long cue‐target intervals in anti‐cue trials. This contrast also yielded more activity in right superior frontal gyrus during the short‐interval trials. This region was shown to be a core region involved in successful response inhibition and specifically action restraint (Aron et al., 2004; Dambacher et al., 2014a, 2014b; Swick et al., 2011). Research using noninvasive brain stimulation further emphasized the role of the right superior frontal gyrus in response restraint (Dambacher et al., 2014c). This is in line with the interpretation that cognitive response inhibition mechanisms play a role in the anti‐cue task. Whether, however, the reported activation reflects the involvement of a very specific uniquely response inhibition related network or rather of a more general action updating network remains to be discussed (Verbruggen, Aron, Stevens, & Chambers, 2010).

Fronto‐striatal networks were suggested to account for high‐level cognitive as opposed to low‐level habitual inhibition which is mostly monitored by the basal ganglia (Jahanshahi, Obeso, Rothwell, & Obeso, 2015). This is not to say that the basal ganglia did not play a role in the present anti‐cue task. Our data showed that the putamen was more active in the short anti‐cue condition than in the long anti‐cue condition, which suggests early suppression of competing response options to facilitate response selection. Interestingly, the putamen was also more active in the long pro‐cue condition than in the long anti‐cue condition. One possible interpretation of this finding is that inhibition may also be necessary to prevent the premature execution of an already selected response, especially with longer preparation intervals (Duque, Lew, Mazzocchio, Olivier, & Ivry, 2010).

### 4.3 Motor preparation

The role of pre‐SMA in proactive motor preparation was repeatedly emphasized in research on animals (Fujii, Mushiake, & Tanji, 2002; Halsband, Matsuzaka, & Tanji, 1994) and humans (Chen et al., 2010; Jaffard et al., 2008; Stuphorn & Emeric, 2012; Wardak, 2011). It was especially shown to be causally relevant to the organization of action sequences (Kennerley, Sakai, & Rushworth, 2004). Interestingly, in our study, such activation was predominantly detected when contrasting pro‐cue trials against anti‐cues trials with long cue‐target interval. This is in line with the notion that when a target is preceded by a timely valid cue, the motor system has plenty of time to prepare and can allocate all necessary resources to trigger the correct effector. This preparation is then mirrored in reaction time benefits. Furthermore, pre‐SMA is involved in the setting and adjusting of response thresholds. The increased activity in pro‐ versus anti‐cue trials at long cue‐target intervals might be elicited, because the pre‐SMA has more time to set more efficient response thresholds when the cue is congruent with the response to be made. This could also be linked to potential connectivity with subcortical structures (e.g., putamen) and the interaction of activity in such areas.

### 4.4 Monitoring ongoing cognitive processes

Our results also show that anti‐cue trials trigger activation in the cingulate cortex. This activation was again more pronounced for the short compared to the long cue‐target interval. The rostral anterior and posterior cingulate has previously been linked specifically to error (error, in this context referring to the preparation of the incorrect effector) processing in a go/nogo paradigm (Menon, Adleman, White, Glover, & Reiss, 2001), suggesting that the human error monitoring system substantially overlaps with networks which have been related to action planning. Klein et al. (2007) elaborate that activation in insular cortex impacted the conscious percept of an error. The combination of cingulate activity (error signaling) and insula activity (response inhibition) seems crucial to initiate adjustment reactions (Klein et al., 2007), as is needed in the anti‐cue task.

## 5 Conclusion

While the only previously available data on neural correlates involved in the anti‐/pro‐cue paradigm pointed to involvement of the basal ganglia (Adam et al., 2011), our whole‐brain imaging approach revealed a bigger picture. The multitude of neural networks we detected demonstrates that proactive motor mechanisms involve several widespread and well‐known cognitive networks tuned by context (pro‐cues vs. anti‐cues) and temporal dynamics (short vs. long cue‐target intervals). A key finding of this study was the limited neural recruitment evoked by the spatially congruent pro‐cues compared to the much more elaborate neural activation patterns elicited by the spatially incongruent anti‐cues, the latter drawing on neural resources hypothesized to be related to attention shifts, error monitoring, and response inhibition. This set of findings fits with the dual‐route framework of response selection, which distinguishes between a fast, automatic, direct route, and a slower, voluntary, indirect route that draws upon executive resources. Furthermore, cue‐target interval appeared to be a strong modulator of the behavioral and neural signatures, indicating that proactive cognitive control is flexible and contingent on preparation time. Further research is now required to start disentangling the various brain correlates and networks at hand, and their interaction. Fortunately, our adapted paradigm has proved sensitive and robust enough to allow for such studies.

## Conflict of Interest

None declared.

## Note

1 The participant in question did not revert to the incorrect response buttons due to compromised task‐understanding or compliance but because of a technical misunderstanding. He/she shifted one entire hand on the fMRI‐response‐button‐box and as not all buttons on the box were coded for (only the four task‐required buttons were), the behavioral responses could not be recoded to be fully analyzed. The participant did, however, perform perfectly in experiment 1 as well as in another behavioral test following the fMRI (to ensure his/her understanding of the task).
